# Chromosome 1p Loss and 1q Gain for Grading of Meningioma

**DOI:** 10.1001/jamaoncol.2025.0329

**Published:** 2025-04-03

**Authors:** Alexander P. Landry, Justin Z. Wang, Vikas Patil, Jeff Liu, Chloe Gui, Yosef Ellenbogen, Andrew Ajisebutu, Leeor Yefet, Qingxia Wei, Olivia Singh, Julio Sosa, Sheila Mansouri, Aaron A. Cohen-Gadol, Ghazaleh Tabatabai, Marcos Tatagiba, Felix Behling, Jill S. Barnholtz-Sloan, Andrew E. Sloan, Silky Chotai, Lola B. Chambless, Alireza Mansouri, Serge Makarenko, Stephen Yip, Felix Ehret, David Capper, Derek S. Tsang, Jennifer Moliterno, Murat Gunel, Pieter Wesseling, Felix Sahm, Kenneth Aldape, Andrew Gao, Gelareh Zadeh, Farshad Nassiri

**Affiliations:** 1 MacFeeters Hamilton Neuro-Oncology Program, Princess Margaret Cancer Centre, University Health Network and University of Toronto, Toronto, Ontario, Canada; 2 Division of Neurosurgery, Department of Surgery, University of Toronto, Toronto, Ontario, Canada; 3 Princess Margaret Cancer Centre, University Health Network, Toronto, Ontario, Canada; 4 Department of Neurological Surgery, Keck School of Medicine, University of Southern California, Los Angeles; 5 German Cancer Consortium, Tübingen University Hospital, Tübingen, Germany; 6 Cluster of Excellence “Image Guided and Functionally Instructed Tumor Therapies,” Eberhard Karls University Tübingen, Tübingen, Germany; 7 Department of Neurology and Interdisciplinary Neuro-Oncology, Center for Neuro-Oncology, Comprehensive Cancer Center, Hertie Institute for Clinical Brain Research, Eberhard Karls University Tübingen, Tübingen, Germany; 8 Department of Neurosurgery, Center for Neuro-Oncology, Comprehensive Cancer Center, Eberhard Karls University Tübingen, Tübingen, Germany; 9 Trans Divisional Research Program, Division of Cancer Epidemiology and Genetics, National Cancer Institute, Bethesda, Maryland; 10 Center for Biomedical Informatics & Information Technology, National Cancer Institute, Bethesda, Maryland; 11 Department of Neurological Surgery & Piedmont Brain Tumor Center, Atlanta, Georgia; 12 Department of Neurosurgery, Vanderbilt University Medical Center, Nashville, Tennessee; 13 Department of Neurosurgery, Penn State Milton S. Hershey Medical Center, Hershey, Pennsylvania; 14 Division of Neurosurgery, University of British Columbia, Vancouver, British Columbia, Canada; 15 Department of Pathology & Laboratory Medicine, Faculty of Medicine, University of British Columbia, Vancouver, British Columbia, Canada; 16 Charité – Universitätsmedizin Berlin, Corporate Member of Freie Universität Berlin and Humboldt-Universität zu Berlin, Department of Radiation Oncology, Berlin, Germany; 17 German Cancer Consortium, Charité – Universitätsmedizin Berlin, Berlin, Germany; 18 Charité – Universitätsmedizin Berlin, Corporate Member of Freie Universität Berlin and Humboldt-Universität zu Berlin, Department of Neuropathology, Berlin, Germany; 19 Radiation Medicine Program, Princess Margaret Cancer Centre, Toronto, Ontario, Canada; 20 Department of Neurosurgery, Yale School of Medicine, New Haven, Connecticut; 21 Department of Pathology, Amsterdam University Medical Center, Amsterdam, the Netherlands; 22 Princess Máxima Center for Pediatric Oncology, Utrecht, the Netherlands; 23 Department of Neuropathology, Heidelberg University, Heidelberg, Germany; 24 Center for Cancer Research, National Cancer Institute, Bethesda, Maryland; 25 Division of Laboratory Medicine and Pathobiology, University Health Network, Toronto, Ontario, Canada

## Abstract

**Importance:**

The World Health Organization (WHO) classification of central nervous system tumors (CNS) grading for meningioma was updated in 2021 to include rare molecular features, namely homozygous deletions of *CDKN2A* or *CDKN2B* and *TERT* promotor alterations. Previous work, including the cIMPACT-NOW statement, has discussed the potential value of including chromosomal copy number alterations to help refine the current grading system.

**Objective:**

To identify chromosomal copy number alterations that could be used to improve the current CNS WHO grading of meningioma.

**Design, Setting, and Participants:**

In this cohort study, patients with surgically treated meningioma were followed-up until recurrence or progression of disease or death. Chromosomal copy number alterations were then correlated with progression-free survival (PFS) to identify new outcome biomarkers. This study included patients with a histopathological diagnosis of meningioma from multiple institutions in Canada, the US, and Germany, with molecular data collection starting in 2016. Data were analyzed from January to September 2024.

**Exposures:**

All patients underwent surgery for meningioma and a subset underwent radiation therapy.

**Main Outcomes and Measures:**

The main outcome was PFS. Cox regression analysis was used to identify copy number alterations associated with outcomes in the context of WHO grading.

**Results:**

Among 1964 patients with meningioma (1256 female; median [IQR] age, 58 [48-69] years) assessed, loss of chromosome 1p in WHO grade 1 meningiomas was associated with significantly worse outcomes compared with tumors without loss of 1p (median PFS, 5.83 [95% CI, 4.36-∞] years vs 34.54 [95% CI, 16.01-∞] years; log-rank *P* < .001). Outcomes of patients with WHO grade 1 tumors with loss of chromosome 1p were comparable to those of patients with WHO grade 2 tumors (median PFS, 4.48 [95% CI, 4.09-5.18] years). Combined loss of chromosome 1p and gain of chromosome 1q were associated with outcomes that were highly concordant with WHO grade 3 tumors, regardless of initial grade (median PFS: grade 1, 2.23 [95% CI, 1.28-∞] years; grade 2, 1.90 [95% CI, 1.23-2.25] years; grade 3, 2.27 [95% CI, 1.68-3.05] years).

**Conclusions and Relevance:**

These findings highlight a role for cytogenetic profiling in the next iteration of CNS WHO grading, with a specific focus on chromosome 1p loss and 1q gain, suggesting that chromosome 1p loss, in addition to 22q loss, should be added as a criterion for a CNS WHO grade of 2 and addition of 1q gain as a criterion for a CNS WHO grade of 3.

## Key Points

**Question:**

What is the role of chromosomal copy number alterations in meningioma, and how can they be used to inform World Health Organization (WHO) Classification of Tumors of the Central Nervous System (CNS) grading?

**Findings:**

This cohort study of 1964 meningiomas found that chromosome 1p loss in CNS WHO grade 1 meningiomas was associated with outcomes that were highly concordant with CNS WHO grade 2 tumors. The addition of a chromosome 1q gain was associated with outcomes that were highly concordant with CNS WHO grade 3 tumors regardless of initial CNS WHO grade.

**Meaning:**

These findings suggest that inclusion of chromosome 1p loss and 1q gain may inform WHO grading.

## Teaser

This cohort study examines chromosomal copy number alterations that could be used to improve the current World Health Organization grading of meningioma.

## Introduction

Meningioma research has seen significant progress recently, driven largely by a recognition that histopathology alone does not adequately capture the heterogeneity in patient outcomes. To address this, our group and others have identified and characterized meningioma molecular groups with unique biology and outcomes.^1,2,3,4,5^ While nomenclature varies, we defined an immunogenic, NF2–wild type, hypermetabolic, and proliferative group in increasing order of clinical aggressiveness. Despite the value of these groups, implementing them into standard care is limited by cost and resource availability.^6^ Additionally, while the 2021 World Health Organization (WHO) grading of tumors of the central nervous system (CNS) incorporates homozygous deletions of *CDKN2A* or *CDKN2B* and *TERT* promotor alterations,^7^ these are rare and often occur in higher-grade histopathological tumors. Recent work has demonstrated the prognostic value of chromosome-level copy number alterations (CNAs) in meningioma,^8,9^ which are more readily accessible using technologies, such as fluorescence in situ hybridization (although this is limited in its ability to discriminate focal alterations, which may have clinical relevance).^10,11^ In this study, we examine whether patients with WHO grade 1 meningiomas that harbor chromosomal 1p deletion have outcomes similar to those with WHO grade 2 tumors and whether tandem 1p loss with 1q gain is associated with outcomes concordant with a WHO grade of 3.

## Methods

This cohort study was approved by the University Health Network institutional review board. As part of institutional policy and routine surgical consent, patients whose meningiomas were included in this study provided consent for their tumor sample, tumor data and de-identified clinical data to be used for clinical or translational research projects.

Using a multicenter cohort of 1964 meningiomas, DNA methylation was used to infer chromosomal arm-level copy number profile, with a subset (n = 211) validated using whole exome sequencing (eFigure 1 in Supplement 1). Molecular data collection began in 2016. To identify CNAs associated with PFS, a regularized Cox regression model was optimized using individual CNAs, CNS WHO grade, extent of resection, and receipt of adjuvant radiotherapy (RT) as regressors. We selected 10 CNAs, grade, and extent of resection based on nonzero output coefficients. This included multiple previously described alterations (1p, 6p, 10p, 18p, and 18q). We then divided our cohort into patients who received adjuvant RT (339 patients) to identify CNAs also associated with RT response (ie, post-RT PFS).

*P* values were 2-sided, and statistical significance was set at *P* ≤ .05. Data were analyzed from January to September 2024 using R software version 4.4.1 (R Project for Statistical Computing). Further details are provided in eAppendix 1 in Supplement 1.

## Results

**Figure 1.**
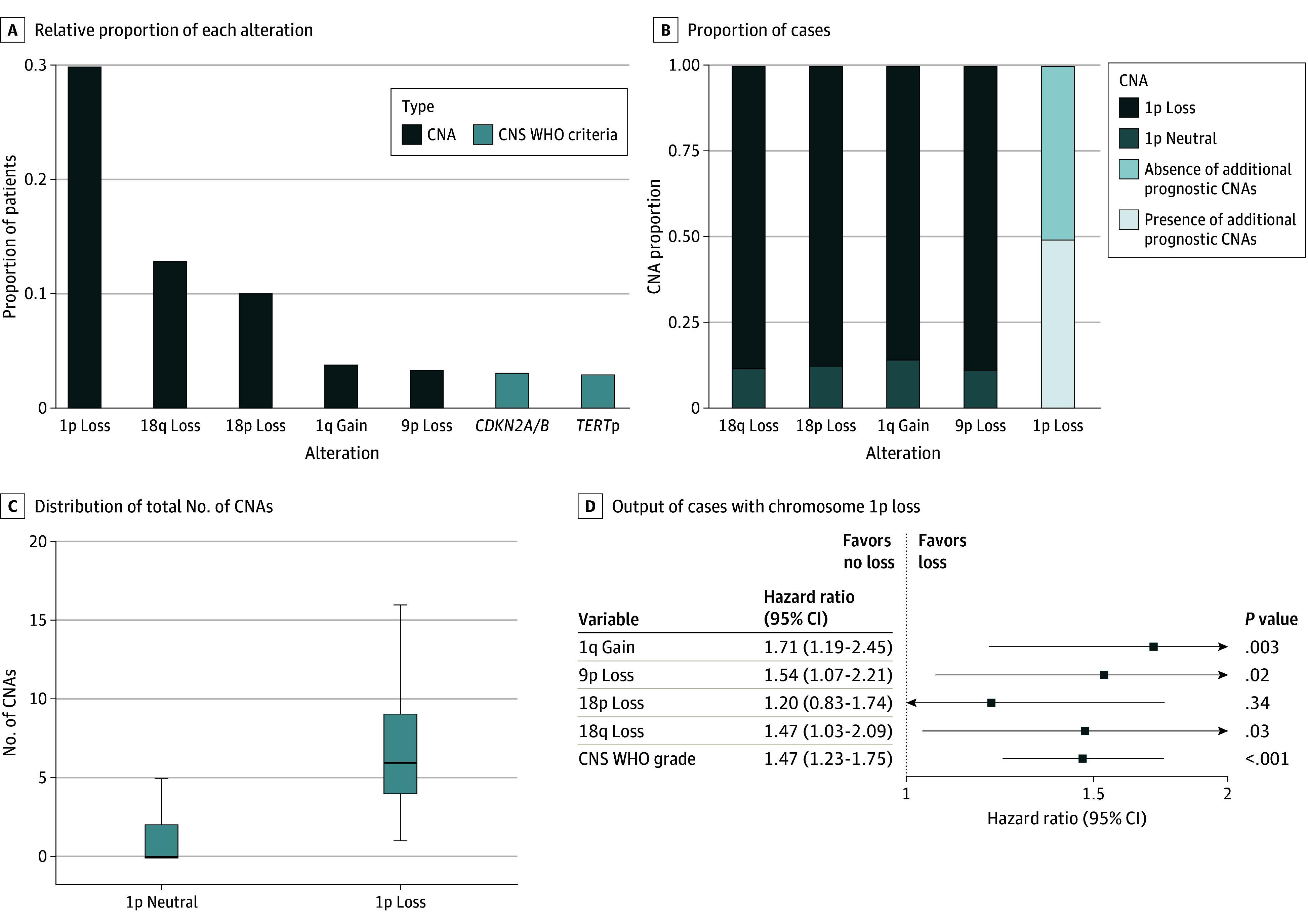
Associations of Multiple Chromosomal Copy Number Alterations (CNAs) With Progression-Free Survival in Meningioma CNS WHO indicates World Health Organization grading for central nervous system tumors; *TERT*p, *TERT* promoter.

A total of 1964 patients with meningioma (1256 female; median [IQR] age, 58 [48-69] years) were assessed. We identified 4 losses (1p, 9p, 18p, and 18q), 2 gains (1q and 21p), and WHO grade as being independently associated with both postsurgical and post-RT PFS. Chromosome 21p was removed from subsequent analysis given its recognized acrocentric nature.^12^ Of remaining CNAs, chromosome 1p loss was most common in our full cohort, present in 576 patients (29.3%), followed by 18q loss (245 patients [12.5%]), 18p loss (188 patients [9.6%]), 1q gain (64 patients [3.4%]), and 9p loss (58 patients [3.0%]). Notably, all were more common than homozygous deletions of *CDKN2A* and/or *CDKN2B* (52 patients [2.6%]) and *TERT* promoter alterations (22 patients [2.5%] of 877 patients with available Sanger sequencing) (Figure 1A). We found that each CNA was highly likely to co-occur with 1p loss (18q loss: 218 patients [89.0%]; 18p loss: 166 patients [88.3%]; 1q gain: 57 patients [86.4%]; 9p loss: 52 patients [89.7%]), whereas approximately half of tumors with 1p loss (294 tumors [51.0%]) did not have any of these additional CNAs (Figure 1B). Meningiomas with 1p loss had a significantly higher total number of CNAs than those without (median [IQR], 7 [4.5-9.5] CNAs vs 0 [0-1] CNAs; Wilcoxon *P* < .001) (Figure 1C). These findings suggest that chromosome 1p loss may be associated with resultant downstream genomic instability, although additional investigation is needed to confirm this.

Given this, we next sought to identify CNAs that are synergistically associated with worse outcomes among 576 patients who had 1p loss already. Using this cohort, we applied multivariable Cox regression to the remaining 5 significant features (1q gain, 9p/18p/18q loss, WHO grade) and found that all but 18p loss remained independently significant (Figure 1D). Given that only 20 patients (1.0%) had grade 1 or 2 and 1p and 9p loss, we chose to focus on 1q gain and 18q loss. Only 1q gain was independently associated with RT response among patients with 1p loss who received adjuvant RT (eFigure 2 in Supplement 1). Furthermore, while tandem 1p and 18q losses were associated with grade 3–like outcomes in patients with grade 2 disease, this was not the case for patients with grade 1 (eFigure 3, eAppendix 2, and eAppendix 3 in Supplement 1).

Only 46 patients (2.3%) had 1p and 22q losses without any other CNAs. Loss of 1p remained significantly associated with PFS in this cohort compared with all 1p neutral tumors in a univariable Cox regression analysis (hazard ratio, 1.75 [95% CI, 1.06-2.90]; *P* = .03). Isolated 1p loss without 22q loss was very rare (15 patients [0.8%]) and did not have a significant association with outcome in an analogous Cox regression. Additional investigation into the role of 1p loss without 22q loss is needed, given the rarity of this event, aligning our conclusions with the recent cIMPACT-NOW statement.^11^

**Figure 2.**
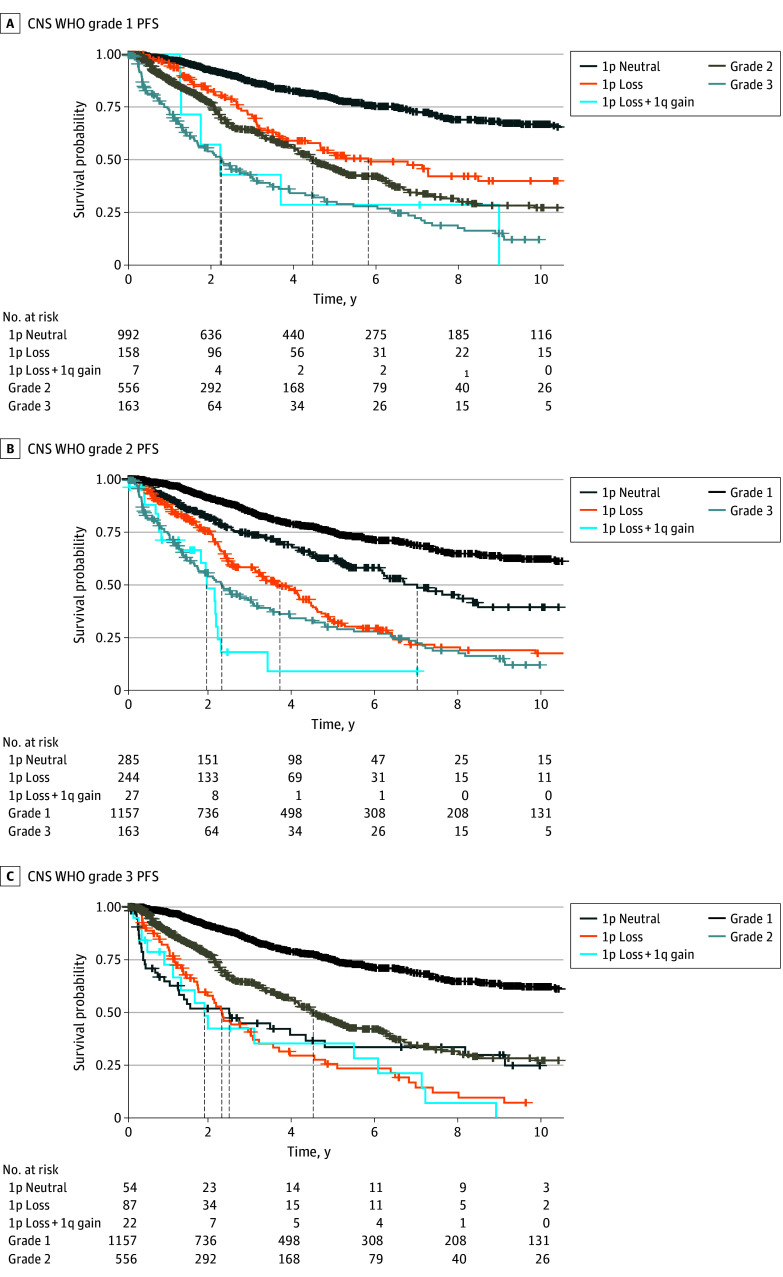
Associations of Chromosome 1p Loss and 1q Gain in Progression-Free Survival (PFS) After Surgery in Meningioma CNS WHO indicates World Health Organization grading for central nervous system tumors. Crosses indicate censoring.

**Figure 3.**
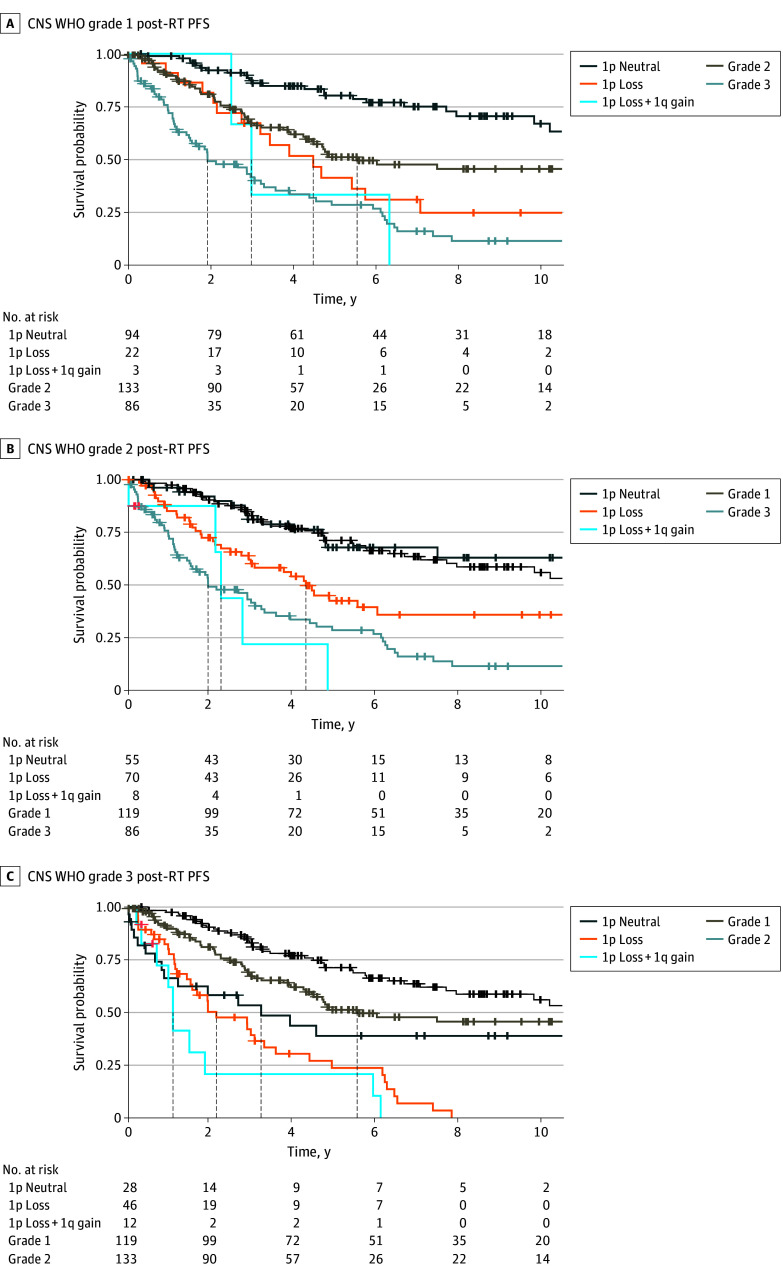
Association of Chromosome 1p Loss and 1q Gain With Adjuvant Radiotherapy (RT) Response in Meningioma CNS WHO indicates World Health Organization grading for central nervous system tumors; PFS, progression-free survival. Crosses indicate censoring.

We next investigated postsurgical PFS stratified by grade and substratified by 1p loss and 1q gain to determine the potential ability of these alterations to refine current grading. Within our cohort, grade 1 disease with 1p loss was associated with similar outcomes to grade 2 overall (median PFS, 5.83 [95% CI, 4.36-∞] years vs 4.48 [95% CI, 4.09-5.18] years). By contrast, patients with grade 1 disease without 1p loss had significantly longer PFS than those with 1p loss (median PFS, 34.54 [95% CI, 16.01-∞] years, log-rank *P* < .001) (Figure 2A) suggesting that 1p loss was associated with grade 2–like outcomes. Patients with grade 1 disease with both 1p loss and 1q gain had PFS similar to patients with grade 3 disease (median PFS, 2.23 [95% CI, 1.28-∞] years vs 2.27 [95% CI, 1.68-3.05] years). Patients with grade 2 disease with 1p loss had shorter PFS than patients with grade 2 disease without 1p loss (median PFS, 3.67 [95% CI, 3.08-4.38] years vs 7.00 [95% CI, 6.11-12.82] years; log-rank *P* < .001), and patients with 1p loss and 1q gain had similar median PFS compared with patients with grade 3 disease (median PFS, 1.90 [95% CI, 1.23-2.25] years vs 2.27 [95% CI, 1.68-3.05] years) (Figure 2B). Stratifying by 1p loss and 1q gain did not significantly impact findings in grade 3 meningiomas (Figure 2C). The same patterns were observed using PFS after adjuvant radiation rather than postsurgical PFS as the outcome of interest (Figure 3; eAppendix 2 in Supplement 1). Notably, only 9 patients had 1q gain without concurrent 1p loss, limiting meaningful conclusions regarding the role of 1q gain in isolation.

## Discussion

In this cohort study, 1p loss and 1q gain were significantly associated with clinical outcomes similar to CNS WHO grade 3 disease, regardless of initial grade. Using these findings to reclassify disease in out cohort would upgrade 156 patients with grade 1 disease (13.3%) to grade 2, 8 patients with grade 1 (0.7%) to grade 3, and 27 patients with grade 2 (4.4%) to grade 3, totaling 191 changes (9.7%) overall. These findings add to the growing literature advocating for the inclusion of cytogenetic profiling into improved CNS WHO grading for meningioma.

### Limitations

This study has some limitations. Further work is needed to assess the clinical importance of focal/partial chromosome 1q gains compared with full 1q gains. Similarly, establishing the value of other technologies that are more widely accessible than DNA methylation profiling, such as fluorescence in situ hybridization, is needed to allow increasingly widespread dissemination of these findings. Furthermore, exploring the clinical impact of intratumoral and regional clonal heterogeneity (eg, cases where only a subset of cells or regions within a tumor have 1p loss and 1q gain) is needed in follow-up to better contextualize our results and understand the potential temporal nature of CNAs in meningioma.

## Conclusions

The findings of this cohort study support the recent c-IMPACT NOW statement advocating for cytogenic profiling in the next iteration of CNS WHO grading of meningiomas. We found that grade 1 meningiomas with chromosome 1p loss had prognosis similar to grade 2, and the addition of 1q gain was associated with grade 3–like outcomes regardless of initial grade.

## Notes

Supplement 1. **eAppendix 1.** Supplemental Methods **eAppendix 2.** Supplemental Results **eAppendix 3.** Supplemental Discussion
eReferences.
**eTable.** Baseline Cohort Characteristics **eFigure 1.** Validation Using Whole-Exome Sequencing **eFigure 2.** Associations of Chromosome 1q With RT Response Among Patients With Chromosome 1p Loss **eFigure 3.** Association of Chromosome 1p Loss and 18q Loss in Meningioma Postsurgical and Post-RT Progression-Free Survival

Supplement 2.
Data Sharing Statement
