# Supplementary material for: Chromosome 1p Loss and 1q Gain for Grading of Meningioma
Source: JAMA Oncol. 2025 Apr 3;11(6):644–9. doi: 10.1001/jamaoncol.2025.0329 (PMC11969356; doi:10.1001/jamaoncol.2025.0329)
Supplement: Supplement 1. — eAppendix 1. Supplemental Methods eAppendix 2. Supplemental Results eAppendix 3. Supplemental Discussion eReferences. eTable. Baseline Cohort Characteristics eFigure 1. Validation Using Whole-Exome Sequencing eFigure 2. Associations of Chromosome 1q With RT Response Among Patients With Chromosome 1p Loss eFigure 3. Association of Chromosome 1p Loss and 18q Loss in Meningioma Postsurgical and Post-RT Progression-Free Survival [file jamaoncol-e250329-s001.pdf]

Supplemental Online Content

Landry AP, Wang JZ, Patil V, et al. Chromosome 1p loss and 1q gain for grading of meningioma. *JAMA Oncol*. Published online April 3, 2025.
doi:10.1001/jamaoncol.2025.0329

eAppendix 1. Supplemental Methods
eAppendix 2. Supplemental Results
eAppendix 3. Supplemental Discussion
eReferences.
eTable. Baseline Cohort Characteristics
eFigure 1. Validation Using Whole-Exome Sequencing
eFigure 2. Associations of Chromosome 1q With RT Response Among Patients With Chromosome 1p Loss
eFigure 3. Association of Chromosome 1p Loss and 18q Loss in Meningioma Postsurgical and Post-RT Progression-Free Survival

This supplemental material has been provided by the authors to give readers additional information about their work.

© 2025 Landry AP et al. *JAMA Oncology*.

Online Methods

Study cohort
Our multicenter cohort consisted of samples from multiple institutions including the University Health Network, Indiana University, Case Western, Northwestern University, Fred Hutchison Cancer Center, Vanderbilt University, Vancouver General Hospital, Baylor (previously published[1]), University of California San Francisco (previously published[2]), Charité – Universitätsmedizin Berlin (previously published[3]), and the University of Tubingen.

Sample processing
The process of acquiring and processing samples has been described previously[4]. In summary, tumor samples from our institution were collected during surgery and frozen at -80°C. Samples from other institutions were either fresh-frozen or formalin-fixed. DNA extraction was carried out using either the DNeasy Blood and Tissue Kit or the QIAamp DNA FFPE Tissue Kit from Qiagen.

DNA methylation
We bisulfite converted 250-500ng of DNA using the EZ DNA Methylation Kit, followed by methylation profiling on the Illumina 850k EPIC array. Raw data was preprocessed and ssNoob normalized with the minfi package. Filtering ensued to eliminate probes which failed hybridization (detection p-value > 0.05), probes overlapping with known single-nucleotide polymorphisms, cross-reactive probes, and probes on the X or Y chromosome. Resultant beta-values were used for all subsequent analyses.

Inferred Copy Number Alterations
Chromosome arm-level gains and losses were inferred using the *conumee* package. First, beta values were aligned to the human genome, binned, and segmented by chromosome arm. Median segment values were computed for each arm and thresholded at |0.2| to define a gain (>0.2) or loss (<-0.2), respectively.

TERT promotor sequencing
In addition, amplification of the *TERT* promoter region containing the hotspots C228T and C250T was performed on 694 meningioma tumour samples in the validation cohort. The Platinum SuperFi II PCR Master Mix (ThermoFisher cat no. 12368010) with primers 5'-AGTGGATTCGCGGGCACAGA-3' and 5'- CAGCGCTGCCTGAAACTC-3'[5] was used to produce a 235 base pair amplicon. PCR products were separated by gel electrophoresis to verify a product at the correct size. After purification with the ZR-96 DNA clean-up kit (Zymo Research, cat no. D4018), PCR products were sent for Sanger sequencing at The Centre for Applied Genomics. Chromatograms were analyzed in Geneious Prime, and all mutations were confirmed by sequencing of a second PCR reaction.

Exome sequencing and CNA inference
A total of 211 samples from the University Health Network underwent whole exome sequencing. As previously described[4], exome libraries were prepared using 100ng of DNA from tumour or matched normal DNA (plasma) prior to pair-ended sequencing on a HiSeq 2500 platform. Fastq files were aligned to the hg19 genome using BWA-MEM[6] (v.0.7.12). Allele-specific copy

© 2025 Landry AP et al. *JAMA Oncology*.

number profiles were computed using Sequenza (v2.1.2) and CNVkit[7] (v0.9.6). Gene-level log2ratio data were binned by chromosome arm and the median value of each arm thresholded at |0.5| to define a chromosome arm gain or loss.

Survival analysis
To determine prognostic chromosome arm-level CNAs, the segmented value of each chromosome arm (-1, 0, or 1) was input into a multivariable elastic net regularized Cox regression model along with CNS WHO grade, extent of resection (gross-total vs subtotal resection), and receipt of adjuvant radiotherapy (yes/no). Time to progression/recurrence, defined as radiological growth resulting in change to clinical management, was used as the outcome variable. Alpha and lambda hyperparameters were optimized using 5-fold cross-validation. Regressors with non-zero coefficients in the final model were considered important features. This process was repeated both on the full cohort, with post-surgical PFS as the outcome variable, as well as the subset of cases which received adjuvant RT, with post-RT PFS as the outcome variable. Kaplan Meier analysis was then applied to compare outcome (PFS) between each CNS WHO grade stratified by the presence/absence of prognostic CNAs using pairwise log-rank test. Alpha was chosen to be 0.05 throughout this study.

Statistical analysis
All analysis reported in this manuscript was performed using the open-source computational platform R[8] (version 4.4.1).

© 2025 Landry AP et al. *JAMA Oncology*.

Online Results

Chromosome 1p loss with 1q gain is associated with grade 3-like RT responses
Kaplan Meier analysis demonstrated that CNS WHO grade 1 cases with 1p loss had similar median post-RT PFS (4.48 [95%CI 2.75-Inf] years) compared to CNS WHO grade 2 cases (5.55 [4.44-Inf] years); only 3 CNS WHO grade 1 cases had both 1p loss and 1q gain (**Figure 3A**). CNS WHO grade 2 cases without 1p loss had very similar post-RT PFS compared to CNS WHO grade 1 cases (median PFS not reached in either case, 95%CI 7.48-Inf and 7.95-Inf years, respectively), and those with 1p loss had significantly shorter PFS (median 4.30 [2.96-Inf] years) than those without (log-rank p = 0.004), but longer than CNS WHO grade 3 cases (1.93 [1.51-3.30] years, log-rank p = 0.007). Importantly, CNS WHO grade 2 cases with both 1p loss and 1q gain (median PFS 2.24 [2.11-Inf] years) had similar outcomes to CNS WHO grade 3 cases (**Figure 3B**). No effect was noted among CNS WHO grade 3 cases (**Figure 3C**).

CNS WHO grade 2 meningiomas with 1p and 18q losses have grade 3-like behaviour
Kaplan Meier analysis demonstrated that CNS WHO grade 1 cases with 1p loss had similar PFS (median 7.28 [3.65-Inf] years) to both CNS WHO grade 1 cases with 1p and 18q losses (4.84 [3.10-10.74] years) and CNS WHO grade 2 cases overall (4.48 [4.09-5.18] years). PFS among CNS WHO grade 3 cases (median 2.27 [1.68-3.05] years) was significantly shorter than CNS WHO grade 1 cases with 1p and 18q loss (log-rank p = 0.005, **Supplemental Figure 3A**). Similarly, median post-RT PFS was remarkably similar between CNS WHO grade 1 cases with 1p loss (3.91 [3.21-Inf] years) and CNS WHO grade 1 cases with both 1p and 18q losses (3.74 [2.50-Inf] years, **Supplemental Figure 3B**), suggesting that 18q loss does not confer prognostic value on top of 1p loss in CNS WHO grade 1 meningioma. Among CNS WHO grade 2 cases, those with both 1p and 18q losses had significantly shorter PFS (median 2.42 [2.09-3.61] years) than those with 1p loss (4.03 [3.19-5.00] years, log-rank p = 0.009) but similar median PFS to CNS WHO grade 3 cases (2.27 [1.68-3.05] years). Similarly, post-RT PFS was significantly shorter in CNS WHO grade 2 cases with both 1p and 18q losses (median 2.76 [1.63-4.49] years) than those with only 1p loss (11.77 [4.25-Inf] years, log-rank p = 0.002) and similar to CNS WHO grade 3 cases (median 1.93 [1.51-3.30] years). CNS WHO grade 3 cases with both 1p and 18q losses had shorter PFS (median 1.61 [1.27-2.97] years) than those with only 1p loss (median 2.90 [2.05-6.36] years, log-rank p = 0.005), but not shorter than CNS WHO grade 3 cases without 1p loss (median PFS 2.44 [1.21-8.17] years), and no statistically significant differences were noted in the RT cohort.

© 2025 Landry AP et al. *JAMA Oncology*.

Online Discussion

In this study, we leverage a large multi-centered cohort to demonstrate how chromosomal copy-number alterations can be used to inform CNS WHO grading of meningiomas. Using over 1900 samples, we found that CNS WHO grade 1 cases with 1p loss are associated with CNS WHO grade 2-like clinical outcomes, both in terms of post-surgical PFS and post-RT PFS (RT response). We also demonstrate, for the first time, that 1q gain in tandem with 1p loss imparts CNS WHO grade 3-like clinical behaviour regardless of current CNS WHO grade. Notably, we also show that CNS WHO grade 2 meningiomas with both 1p and 18q losses also portend CNS WHO grade 3-like outcomes.

It is noteworthy that multiple approaches yielded 1q gain as a synergistically prognostic CNA on top of 1p loss. In addition to the elastic net model presented, we also constructed a 2-layer multivariable Cox regression approach wherein each individual CNA was first assessed in a standard Cox regression model adjusting for CNS WHO grade, EOR, and receipt of adjuvant RT. All independently prognostic CNAs in these models ($n = 21$) were then input into a larger multivariable Cox regression which was also adjusted for CNS WHO grade, EOR, and receipt of adjuvant RT. In this formulation, only 1p loss and 1q gain remained independently predictive of both post-surgical and post-RT PFS ($p<0.05$).

Loss of chromosome 1p and gain of 1q has been associated with other molecular nomenclature described in meningioma. We have previously shown that 1p loss is almost universal among both hypermetabolic (MG3) and proliferative (MG4) meningioma molecular groups. By contrast, 1q gain is highly specific to MG4 cases, present in 34% of proliferative cases but only 2% of hypermetabolic cases[4]. We would therefore contend that meningiomas with 1p loss and 1q gain in fact represent proliferative cases, and that the integration of these markers into the CNS WHO criteria represents an important avenue through which modern molecular discoveries can begin to permeate the standard of care.

Further work is needed to assess the clinical importance of focal/partial chromosome 1q gains compared to full 1q gains. Similarly, establishing the value of other technologies which are more widely accessible that DNA methylation profiling (though which may not detect such focal gains), such as fluorescence in situ hybridization, is needed to allow increasingly widespread dissemination of these findings. Finally, exploring the clinical impact of intra-tumoural and regional clonal heterogeneity (i.e. cases where only a subset of cells or regions within a tumour have 1p loss and 1q gain, for example) is needed in follow-up to better contextualize our results and understand the potential temporal nature of copy number alterations in meningioma.

© 2025 Landry AP et al. *JAMA Oncology*.

eReferences

1. Bayley, J. C. *et al.* Multiple approaches converge on three biological subtypes of meningioma and extract new insights from published studies. *Sci Adv* **8**, (2022).
2. Choudhury, A. *et al.* Meningioma DNA methylation groups identify biological drivers and therapeutic vulnerabilities. *Nat Genet* **54**, 649–659 (2022).
3. Ehret, F. *et al.* Clinical implications of DNA methylation-based integrated classification of histologically defined grade 2 meningiomas. *Acta Neuropathol Commun* **12**, 74 (2024).
4. Nassiri, F. *et al.* A clinically applicable integrative molecular classification of meningiomas. *Nature* **597**, 119–125 (2021).
5. Spiegl-Kreinecker, S. *et al.* *TERT* promoter mutations are associated with poor prognosis and cell immortalization in meningioma. *Neuro Oncol* **20**, 1584–1593 (2018).
6. Li, H. & Durbin, R. Fast and accurate short read alignment with Burrows–Wheeler transform. *Bioinformatics* **25**, 1754–1760 (2009).
7. Talevich, E., Shain, A. H., Botton, T. & Bastian, B. C. CNVkit: Genome-Wide Copy Number Detection and Visualization from Targeted DNA Sequencing. *PLoS Comput Biol* **12**, e1004873 (2016).
8. R Development Core Team. R: A Language and Environment for Statistical Computing. *R Foundation for Statistical Computing Vienna Austria* **0**, {ISBN} 3-900051-07-0 (2016).

© 2025 Landry AP et al. *JAMA Oncology*.

Supplemental Table 1: Baseline cohort characteristics.

	Full cohort (n = 1964)	RT cohort (n = 339)
Median Age (Q1-Q3)	58 (47.5-68.5)	58 (47-69)
Sex		
Male	652/1908 (34.2%)	125/334 (37.4%)
Female	1256/1908 (65.8%)	209/334 (62.6%)
Tumour status		
Primary	1310/1607 (81.5%)	176/270 (65.2%)
Recurrent	297/1607 (18.5%)	94/270 (34.8%)
WHO Grade		
1	1174/1959 (59.9%)	119/339 (35.1%)
2	614/1959 (31.3%)	134/339 (39.5%)
3	171/1959 (8.7%)	86/339 (25.4%)
Extent of resection		
GTR	1294/1858 (69.6%)	166/339 (49.0%)
STR	564/1858 (30.4%)	173/339 (51.0%)

GTR = gross total resection, STR = subtotal resection, RT = radiotherapy

© 2025 Landry AP et al. *JAMA Oncology*.

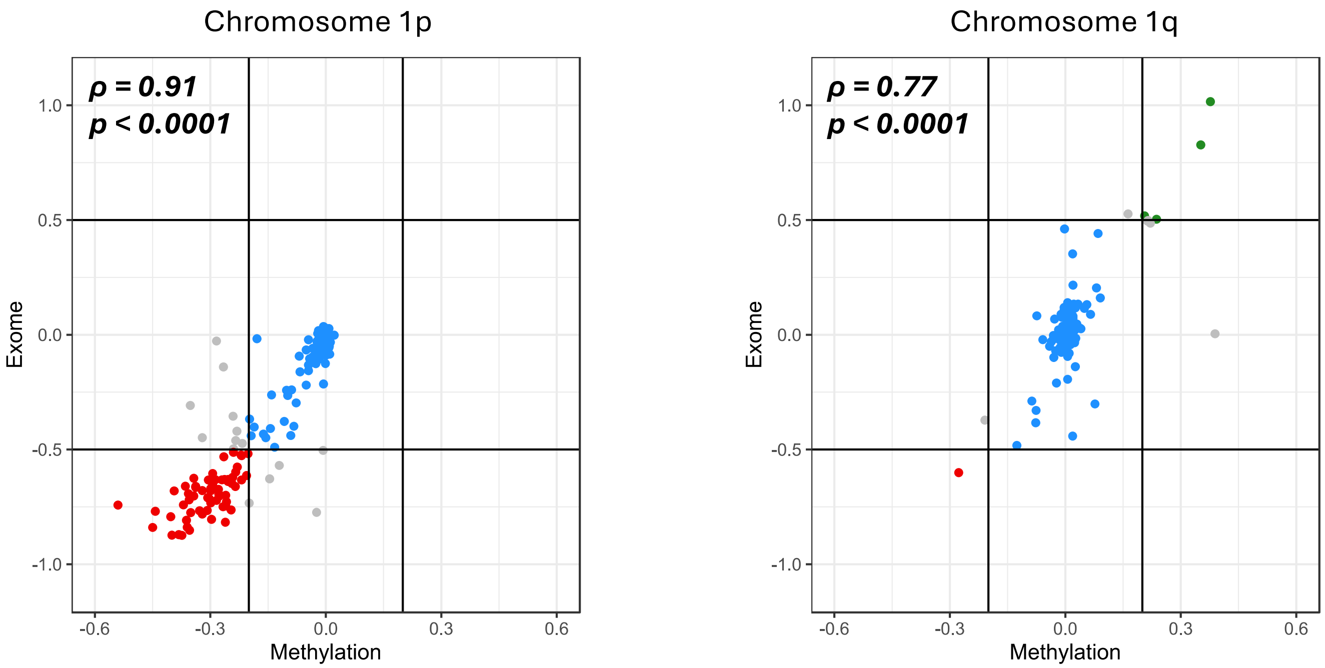

Supplemental Figure 1: Validation using whole exome sequencing. Comparison of chromosome arm median segment log2 ratios using DNA methylation and exome sequencing among samples with both data available. DNA methylation data is thresholded at |0.2| to define a gain or loss, and exome data is thresholded at |0.5| given its increased sensitivity. For chromosome 1p, 197 cases (93%) were concordant between exome and methylation data based on these thresholds. For chromosome 1q, the concordance was 205/211 (97%), which increases to 206/211 (98%) when only considering 1q gains vs 1q non-gains.

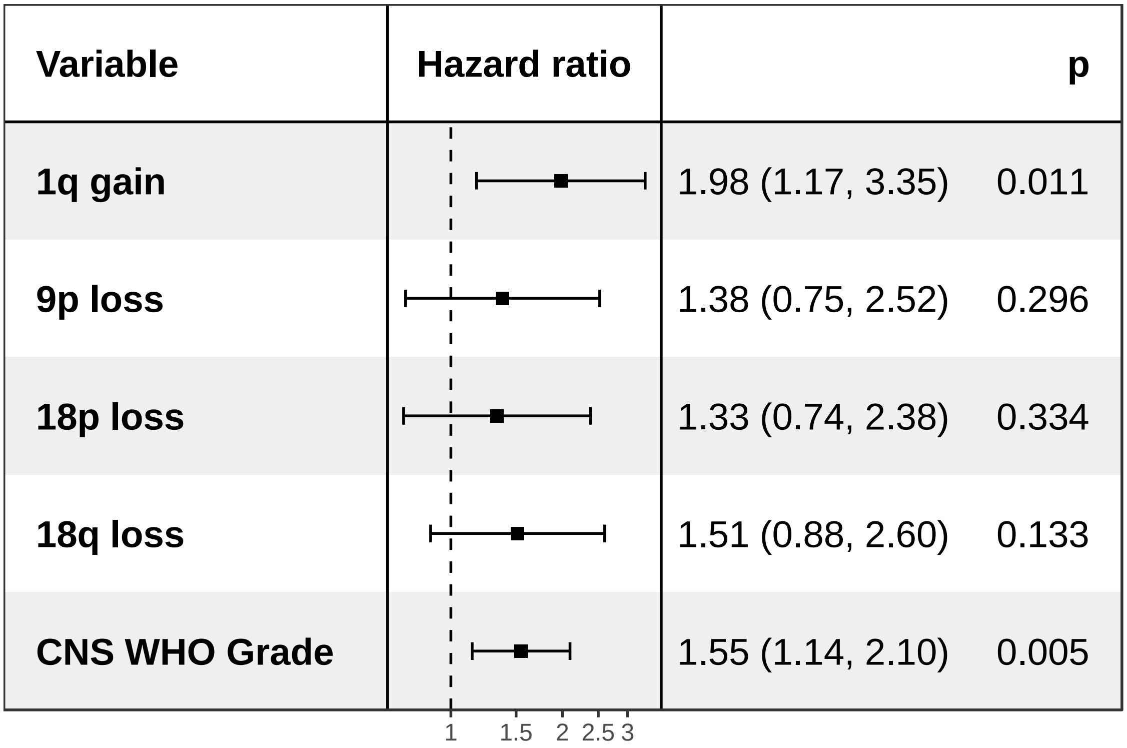

Supplemental Figure 2: Chromosome 1q remains predictive of RT response among cases with chromosome 1p loss. Multivariable Cox regression output of cases with chromosome 1p loss and which underwent adjuvant RT. Post-RT PFS is the output variable in this model.

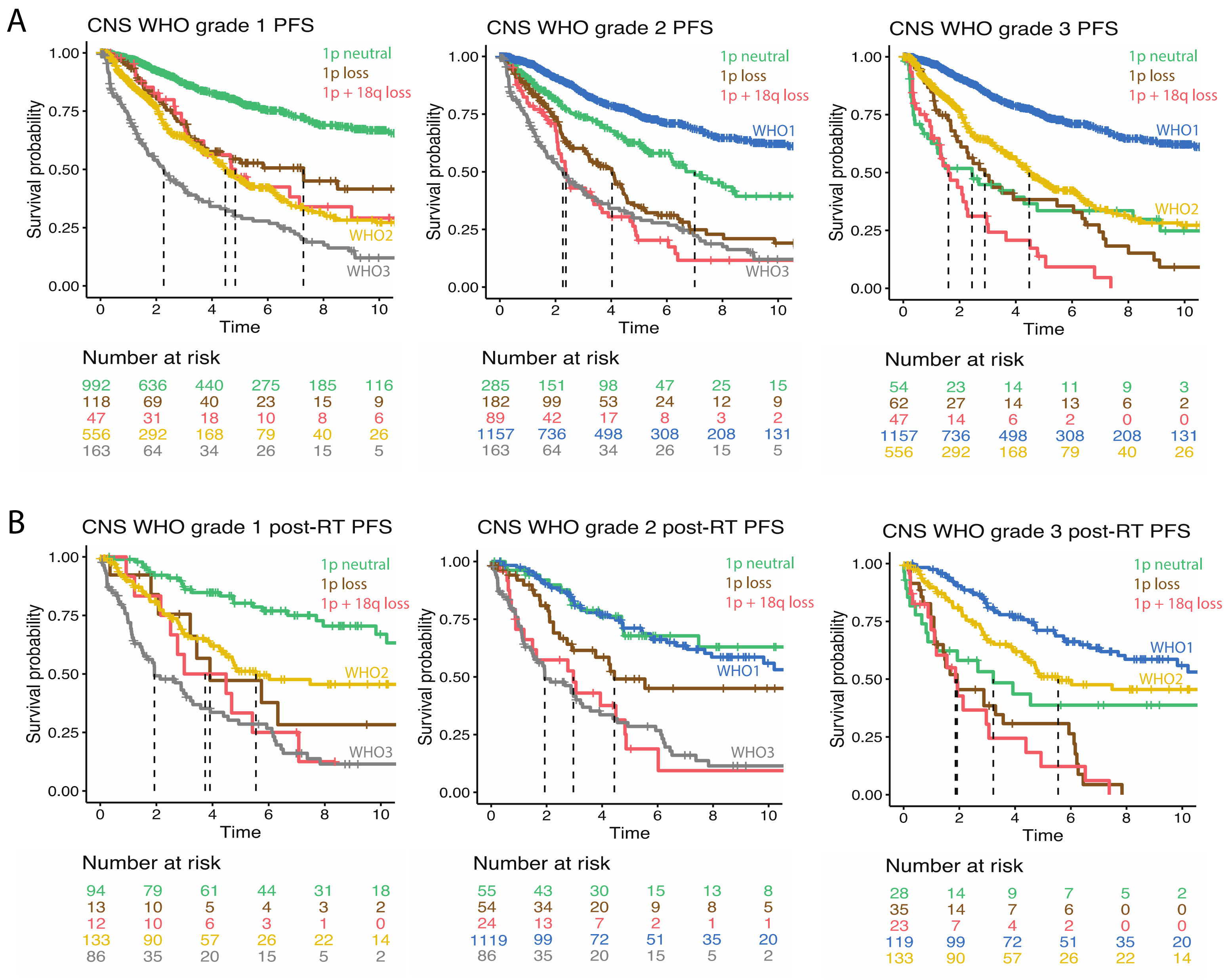
A
CNS WHO grade 1 PFS
CNS WHO grade 2 PFS
CNS WHO grade 3 PFS
1p neutral
1p loss
1p + 18q loss
WHO1
WHO2
WHO3
Survival probability
Time
Number at risk
992 636 440 275 185 116
118 69 40 23 15 9
47 31 18 10 8 6
556 292 168 79 40 26
163 64 34 26 15 5
285 151 98 47 25 15
182 99 53 24 12 9
89 42 17 8 3 2
1157 736 498 308 208 131
163 64 34 26 15 5
54 23 14 11 9 3
62 27 14 13 6 2
47 14 6 2 0 0
1157 736 498 308 208 131
556 292 168 79 40 26
B
CNS WHO grade 1 post-RT PFS
CNS WHO grade 2 post-RT PFS
CNS WHO grade 3 post-RT PFS
94 79 61 44 31 18
13 10 5 4 3 2
12 10 6 3 1 0
133 90 57 26 22 14
86 35 20 15 5 2
55 43 30 15 13 8
54 34 20 9 8 5
24 13 7 2 1 1
1119 99 72 51 35 20
86 35 20 15 5 2
28 14 9 7 5 2
35 14 7 6 0 0
23 7 4 2 0 0
119 99 72 51 35 20
133 90 57 26 22 14

Supplemental Figure 3: The combined influence of chromosome 1p loss and 18q loss in meningioma post-surgical and post-RT progression-free survival. A) Kaplan Meier curves depicting post-surgical PFS stratified by CNS WHO grade, with each CNS WHO grade sub-stratified by CNA status (1p neutral, 1p loss, and 1p loss with 18q loss). B) As (A), but the outcome of interest is post-RT PFS among cases that received adjuvant RT.
