# Supplementary material for: Chromosome 1p Loss and 1q Gain for Grading of Meningioma
Source: JAMA Oncol. 2025 Apr 3;11(6):644–9. doi: 10.1001/jamaoncol.2025.0329 (PMC11969356; doi:10.1001/jamaoncol.2025.0329)
Supplement: Supplement 2. — Data Sharing Statement [file jamaoncol-e250329-s002.pdf]

Data Sharing Statement

Data
Data available: Yes
Data types: Deidentified participant data
How to access data: This will be made available upon request to the corresponding authors (farshad.nassiri@uhn.ca or gelareh.zadeh@uhn.ca) to comply with our institutions IRB policy on data sharing
When available: With publication

Supporting Documents
Document types: None

Additional Information
Who can access the data: anyone with reasonable request as determined by the corresponding authors
Types of analyses: any purpose
Mechanisms of data availability: with data access agreement
